# Regulation of human papillomavirus E6 oncoprotein function via a novel ubiquitin ligase FBXO4

**DOI:** 10.1128/mbio.02783-24

**Published:** 2024-12-17

**Authors:** Arushi Vats, Luca Braga, Nezka Kavcic, Paola Massimi, Edoardo Schneider, Mauro Giacca, Laimonis A. Laimins, Lawrence Banks

**Affiliations:** 1 Tumour Virology, International Centre for Genetic Engineering and Biotechnology, Trieste, Italy; 2 Department of Microbiology-Immunology, Northwestern University Feinberg School of Medicine, Chicago, Illinois, USA; 3 Functional Cell Biology Laboratory, International Centre for Genetic Engineering and Biotechnology, Trieste, Italy; 4 School of Cardiovascular & Metabolic Medicine and Sciences, King's College London British Heart Foundation Centre, London, United Kingdom; The University of North Carolina at Chapel Hill, Chapel Hill, North Carolina, USA

**Keywords:** HPV, E6, E6AP, FBXO4

## Abstract

Previous studies have shown that E6 interacts with the E6-associated protein (E6AP) ubiquitin-protein ligase and directs its ubiquitylation activity toward several specific cellular proteins, one of the most important of which is p53. Interestingly, E6AP not only aids in the E6-directed degradation of cellular substrates but also stabilizes the E6 protein by protecting it from proteasome-mediated degradation. However, there is no information available about the ubiquitin ligases that regulate the stability and activity of the human papillomavirus (HPV) E6 oncoprotein in the absence of E6AP. Therefore, to identify these novel ubiquitin ligases, we performed high-throughput human siRNA library screen against ubiquitin ligases in clustered regularly interspaced palindromic repeat (CRISPR)-edited E6AP-knockout human embryonic kidney (HEK) 293 cells, stably expressing green fluorescent protein (GFP)-tagged HPV-18E6. We found a number of ubiquitin ligases that increase the expression of GFP-tagged 18E6 upon their knockdown in the absence of E6AP. Upon validation of the interaction of 18E6 with these ubiquitin ligases in cervical cancer-derived cell lines, we found that the knockdown of ubiquitin ligase F-box protein 4 (FBXO4), together with E6AP knockdown, leads to a dramatic increase in the levels of endogenous HPV-18E6 oncoprotein. Furthermore, our data demonstrate that the combined knockdown of FBXO4 and E6AP not only rescues the protein levels of E6 but also induces high levels of cell death in a p53-dependent manner in the HPV-positive cervical cancer cell line, HeLa. These results indicate a close interplay between FBXO4, E6AP, and p53 in the regulation of cell survival in HPV-positive cervical tumor-derived cells.

**IMPORTANCE:**

E6-associated protein (E6AP)-mediated stabilization of human papillomavirus (HPV) E6 plays a crucial role in the development and progression of cervical and other HPV-associated cancers. This study, for the first time, identifies a novel ubiquitin ligase, FBXO4 that targets the degradation of HPV E6 oncoprotein in the absence of E6AP in cervical cancer-derived cell lines. This may have significant implications for our understanding of HPV-associated cancers by providing deeper insights into the intricate interplay between viral proteins and host cellular machinery and the development of targeted therapies.

## INTRODUCTION

Cervical cancer is one of the most common types of cancer, affecting approximately 500,000 women worldwide. A small subset of human papillomavirus (HPV) types, known as the high-risk HPV types (mainly HPV-16 and -18), are the causative agents of cervical cancer and a large number of other human malignancies. Persistent expression of the HPV E6 and E7 oncoproteins leads to the development of cancer and is a major hallmark of high-risk HPV-induced carcinogenesis. The high-risk HPV E6 oncoprotein is known to contribute to human malignancy by targeting several of its cellular substrates for degradation through the ubiquitin–proteasome pathway (1).

The ubiquitin–proteasome system (UPS) is known to be responsible for maintaining the turnover of many cellular proteins and tumor suppressors that, if not correctly removed from cells, might lead to cellular or DNA damage and, potentially, to malignancy. Many DNA tumor viruses, such as adenovirus, HPV, and SV40, have evolved to exploit the host cell UPS for their own benefit: either degrading tumor-suppressor proteins or regulating their expression to maximize the viral replication. The most common strategy that all these viruses employ is to target the two major tumor suppressors, p53 and pRb, which are known to regulate many cellular pathways involved in controlling tumor progression (2). HPV was the first small DNA tumour virus discovered to use the host cell UPS for its own purposes: directing the degradation of the most studied tumor suppressor, p53, through recruiting a cellular protein, E6-associated protein (E6AP) (3–5). E6 and p53 associate and form a complex with E6AP to degrade either wild-type or mutant forms of p53 (5). In a normal scenario, E6AP has no role in the control of p53 levels, and its ubiquitin ligase activity is strictly regulated. However, loss of E6AP expression has been reported to cause a neurodevelopmental disorder, known as Angelman syndrome (6), while increased expression levels have been shown to be associated with autism spectrum disorders (7).

Previous studies have shown that E6 interacts with the E6AP ubiquitin-protein ligase and directs its ubiquitylation activity toward several specific cellular proteins, one of the most important of which is p53. E6–E6AP together functions as an E3 ligase in conjunction with the E2-conjugating enzymes UbcH5 (8, 9), UbcH6 (10), and UbcH7 (10–12) to target p53 for proteasomal degradation. E6 is also known to associate with HERC2, a HECT-domain E3 ubiquitin ligase, containing NEURL4 and mitogen-activated protein kinase-6 (MAPK6) complex through E6AP (13). Another HECT domain-containing E3 ubiquitin ligase, EDD/UBR5, has been shown to interact with HPV-18E6. Loss of EDD induces the proteolytic activity of the E6/E6AP complex to mediate the degradation of its target proteins, particularly p53 (14). Interestingly, E6AP not only aids in the E6-directed degradation of cellular substrates but also stabilizes the E6 protein levels. Tomaić et al. in 2009 showed that knockdown of E6AP from cervical cancer-derived HeLa cells results in a dramatic decrease in levels of endogenously expressed E6 protein and also results in a decreased E6 half-life (15). This may suggest that the binding of E6 with E6AP masks the site of interaction with other possible ubiquitin ligases that might be involved in the degradation of E6.

Thus, all these studies suggest that another E3 ligase may exist that is capable of targeting the ubiquitination of E6 substrates, as well as E6 itself. However, there is no information available, to date, about the ubiquitin ligases that regulate the stability and activity of E6 in the absence of E6AP. Therefore, to identify these novel ubiquitin ligases, we performed a high-throughput human siRNA library screen against ubiquitin ligases in human embryonic kidney 293 (HEK293) cells that were clustered regularly interspaced palindromic repeat (CRISPR) edited to ablate E6AP expression and stably expressed green fluorescent protein (GFP)-tagged HPV-18E6. We found a number of ubiquitin ligases that, when knocked down, induce a change in the levels of GFP-tagged 18E6 in the absence of E6AP. Upon validation of these results in cervical cancer-derived cell lines, we found that the combined knockdown of both F-box protein 4 (FBXO4) and E6AP leads to a dramatic increase in the levels of endogenous HPV-18E6 oncoprotein. The knockdown of FBXO4 and E6AP not only rescues the protein levels of E6 but also markedly induces p53-dependent cell death in HPV-positive cervical cancer cell lines.

## RESULTS

### E6 oncoprotein undergoes proteasome-dependent degradation in the absence of E6AP

**Fig 1:**
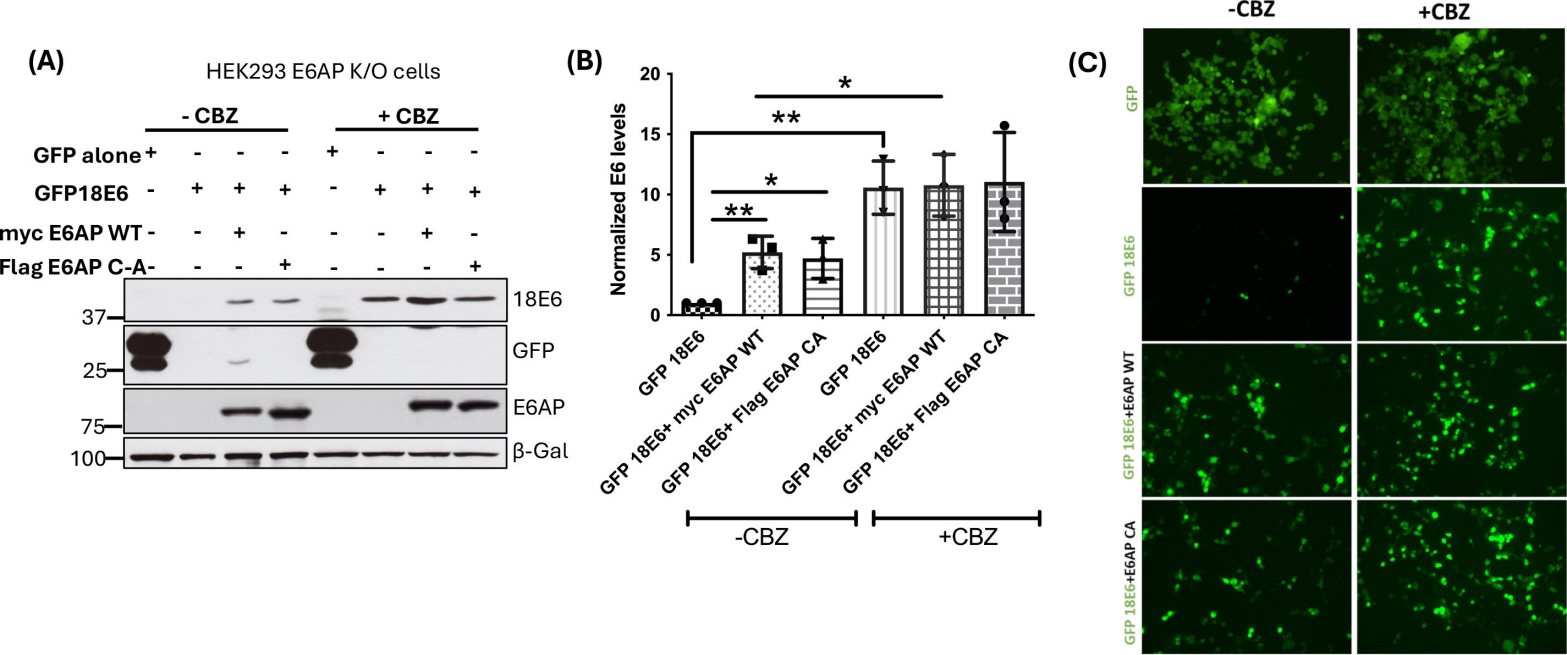
The proteasome inhibitor CBZ inhibits degradation of E6 protein: (**A**) HEK293 E6AP knockout cells were co-transfected with a plasmid expressing GFP-tagged 18E6 (1 µg), together with plasmids expressing either wild-type or catalytically inactive E6AP (2 µg each). After 12 h, the cells were treated with CBZ (2 µM) and incubated for a further 12 h. Cells were then harvested, and E6 protein levels were examined using immunoblotting. β-galactosidase was used as a control for protein loading and transfection efficiency. (**B**) Represents the statistical analysis of the E6 levels in the presence of E6AP and CBZ, from at least three independent experiments; ** and * represent *P*-values <0.05, statistically quantified using Student *t*-test; error bars indicate the standard deviation of the mean. (**C**) Represents the cell fluorescence images of the GFP-tagged 18E6, together with E6AP WT and C-A in the presence and absence of proteasome inhibitor CBZ. A plasmid expressing GFP alone was used as a control.

It has been previously shown that interaction with E6AP stabilizes the HPV E6 oncoprotein by protecting it from proteasomal-mediated degradation, possibly because the binding of E6 with E6AP masks the site of interaction of another possible ubiquitin ligase that might be involved in the degradation of E6 (15). We decided to address this possibility using an HEK293 E6AP-knockout cell line (HEK293 E6AP K/O) as our study model (16). As a preliminary experiment, we co-transfected, into these cells, plasmids expressing functionally active GFP-tagged 18E6 (17) with plasmids expressing either wild-type or catalytically inactive E6AP. Twelve hours after transfection, we blocked proteasomal degradation by treating the cells with the proteasome inhibitor CBZ, then incubated them for a further 12 h, before analyzing protein levels using western blotting and GFP immunofluorescence. In the sample transfected with GFP-tagged 18E6 alone, no GFP was detected; proteasome inhibition clearly rescued 18E6-GFP levels (immunoblotting and GFP fluorescence in Fig. 1A and C, respectively; quantification of three different immunoblotting experiments in Fig. 1B). In the presence of E6AP (wild-type or a catalytically inactive mutant C-A), E6 protein levels were similar in both the untreated samples (no CBZ) and in samples in which the proteasome was inhibited. Treatment with CBZ clearly increased 18E6-GFP levels, in agreement with previous studies (15). Taken together, the above results confirm that E6 undergoes proteasome-dependent degradation in the absence of E6AP, suggesting the involvement of another ubiquitin ligase in mediating its degradation.

### High-throughput library siRNA library screening to identify the potential ubiquitin ligases involved in E6 degradation

To identify the potential ligase involved in the degradation of HPV E6 and its cellular substrates, next, we performed a high-throughput siRNA library screen of all human ubiquitin ligases in HEK293 E6AP K/O cell lines stably expressing GFP-tagged 18E6.

**Fig 2:**
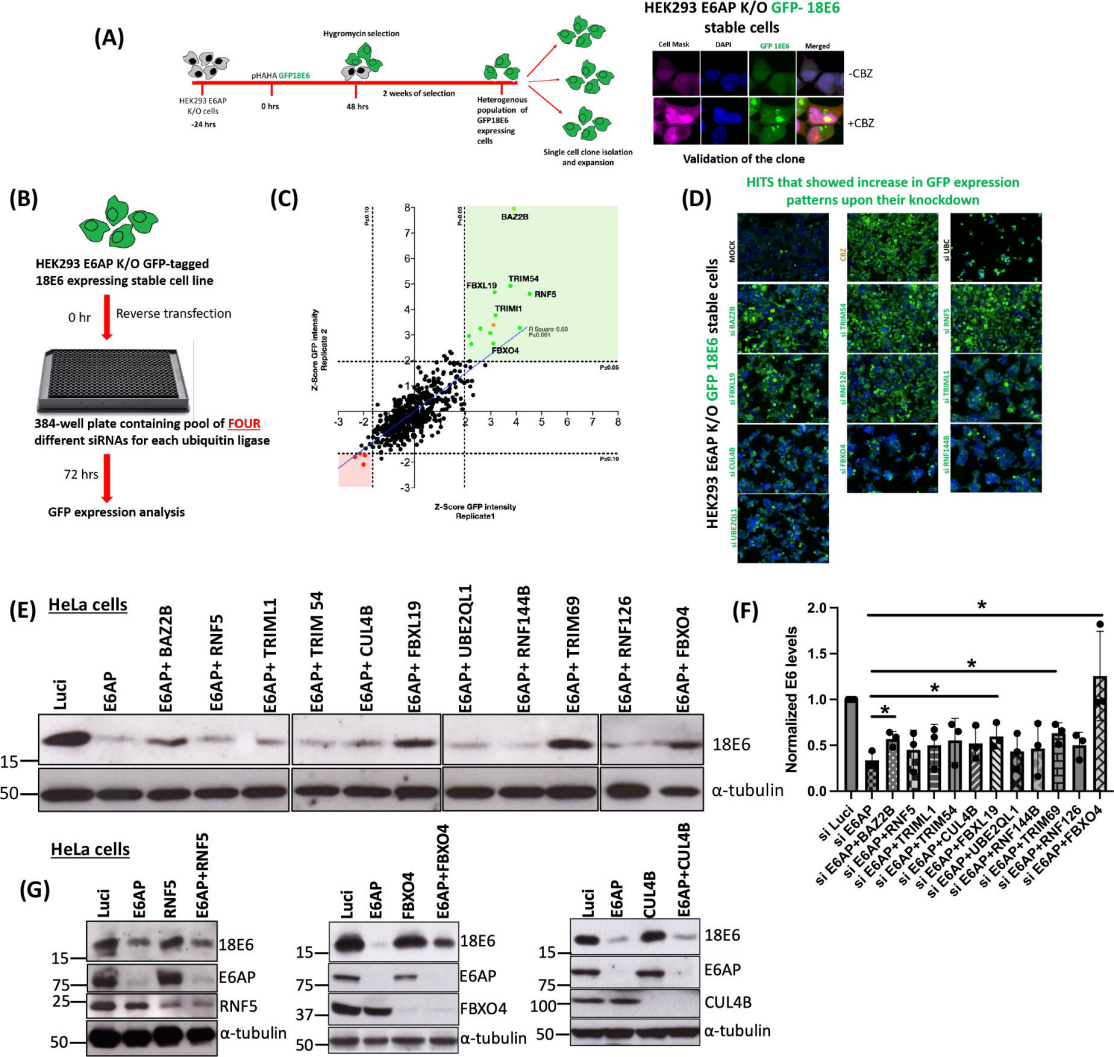
High-throughput human ubiquitin siRNA library screen in HEK293 E6AP K/O cells identifies novel ubiquitin ligases regulating HPV-18E6 stability. (**A**) Schematic diagram showing the generation of HEK293 E6AP knockout cells stably expressing the GFP-tagged 18E6 oncoprotein together with the validation of the stable GFP-tagged 18E6-expressing single-cell clone in the presence and absence of proteasome inhibitor CBZ. (**B**) Represents the experimental outline for a high-throughput siRNA-based screen in HEK293 E6AP K/O cells stably expressing GFP-tagged 18E6, to identify the ubiquitin ligase(s) involved in the degradation of HPV-18E6 oncoprotein. (**C**) Results of the screen: the graphs show the *Z*-score of the log 2 fold change of GFP-positive cells over control in the two replicate screenings (R1 and R2). The dotted lines show a *Z*-score = 1.96; *P* ≤ 0.05 and *Z*-score = −1.65; *P* ≤ 010 (pool of results using four non-targeting siRNAs and mock-transfected cells). The 11 siRNAs in green are those that were the siRNAs that scored significant (*P* ≤ 0.05) in both rounds of screening. The effect of CBZ is shown in yellow. The four siRNAs in red are those that showed significant decrease (*P* ≤ 0.10) in the expression levels of 18E6 GFP upon their knockdown. (**D**) Representative high-content microscopy images showing an increase in GFP-tagged 18E6 protein (shown in green), after depletion of the top 11 cellular ubiquitin-conjugation factors from the screens, when compared with mock, which represents the untreated control cells with basal GFP–18E6 protein levels in HEK293 E6AP K/O cells, CBZ-treated cells are the experimental positive control and siUBC (ubiquitin C gene), toxic upon effective knock-down, is the transfection efficiency control. Scale bar is 50 µm. (**E**) Validation in HeLa cells of the proteins identified by the library screening. HeLa cells were reverse transfected with siRNAs targeting each identified protein, together with siE6AP and siLuciferase (Luci) as control. After 72 h, cells were lysed and protein expression analysis was performed using immunoblotting; α-tubulin was used as a loading control. The blot shown for FBXO4 and RNF126 was run on a different gel. (**F**) Represents statistical analysis of the validation performed in HeLa cells for different ubiquitin ligases identified from the library screening from three independent experiments; * represent *P*-values <0.05, statistically quantified using Student *t*-test; error bars indicate the standard deviation of the mean. (**G**) Western blot analysis confirmed the efficacy of siRNA-mediated knockdowns, demonstrating substantial reductions in protein levels of RNF5, FBXO4, CUL4B, and E6AP in HeLa cells.

Several single-cell clones from the stable cell line generated in HEK293 E6AP K/O expressing GFP-tagged 18E6 were isolated. These clones were then validated by confocal microscopy in the presence and absence of CBZ. In the selected clone, we performed a high-content, fluorescence microscopy-based assay, using a library of siRNAs against factors in the ubiquitin-conjugation system, including E1 and E2 enzymes, and E3 RING and HECT domain ligases (598 target genes, four siRNAs per target, pooled). HEK293 E6AP K/O cells stably expressing GFP-tagged 18E6 were reverse-transfected in 384-well lysine-coated plates with each siRNA (efficiency of transfection > 85%). After 72 h, the cells were fixed, the nuclei were counterstained with Hoechst 33342, and the whole cells were stained with a cell mask (which stains both cytoplasm and nuclei), and GFP fluorescence was analyzed by high-content microscopy (workflow shown in Fig. 2B). Two independent replicates of the screen were conducted; the replicates showed good reproducibility (Spearman *r*  =  0.60; Fig. 2C). The results of the two screens for the 503 siRNAs that did not impair cell viability were expressed as *Z*-score of log2-fold over mock control (shown in Fig. S1). Treatment of the cells with proteasome inhibitor CBZ and with a non-targeting (NT) siRNA (both performed in quadruplicate) served as internal controls. Upon analysis, we observed that the ablation of RNF5, BAZ2B, TRIM54, TRIML1, and FBXL19 resulted in a substantial increase in the levels of GFP-tagged 18E6 protein, while ablation of CUL4B, RNF126, RNF144B, TRIM69, FBXO4, and UBE2QL1 gave a modest increase, as shown in Fig. 2C and D. Intriguingly, we also found four ubiquitin ligases, ASB1, MEX3C, ZNF547, and ZNRF3 (shown as red dots in Fig. 2C), which, when silenced, led to a further decrease in the protein levels of GFP–18E6.

Since the screen was performed in HEK293 E6AP K/O cells, which are derived from human embryonic kidney, we validated the 11 proteins identified by the screen in the cervical cancer-derived cell line HeLa. We reverse-transfected HeLa cells with the siRNAs against each of the shortlisted ubiquitin ligases, together with siE6AP and siLuci (luciferase) as controls. The results obtained are shown in Fig. 2E; as expected, the knockdown of E6AP reduced the levels of E6, in agreement with previous studies (15), when compared with the control siLuci treatment. Upon analysis, we observed that only the knockdown of FBXL19, FBXO4, and TRIM69, together with E6AP, significantly rescued 18E6 protein levels, while siBAZ2B + E6AP and siCUL4B + E6AP showed only a modest effect, when compared with siE6AP. The knockdown of the other proteins identified in the screen (together with E6AP), including RNF5, TRIM54, UBE2QL1, RNF144B, and RNF126, had no effect on the levels of 18E6. We verified the efficacy of the knockdowns for several ubiquitin ligases for which reliable antibodies were available. As demonstrated in Fig. 2G, Western blot analysis confirmed efficient depletion of RNF5, CUL4B, FBXO4, and E6AP in HeLa cells following siRNA treatment. Notably, rescue of HPV E6 levels was observed exclusively in cells with concurrent knockdown of FBXO4 and E6AP, while knockdowns of other tested ubiquitin ligases did not elicit a similar effect on E6 protein levels.

**Fig 3:**
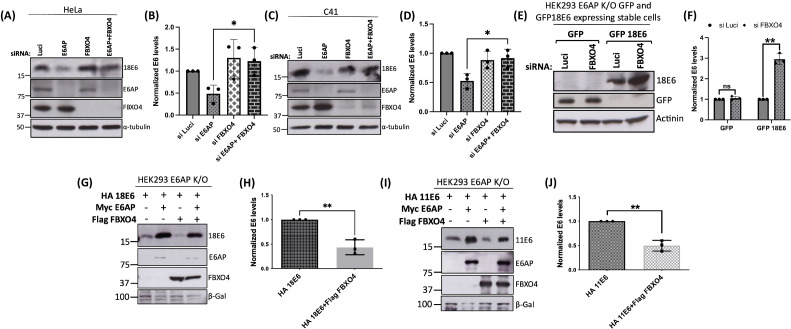
FBXO4 degrades HPV E6 in the absence of E6AP. HeLa (**A**) and C4-1 (**C**) cells were reverse transfected with the siLuci (control), siE6AP, siFBXO4, and siE6AP + siFBXO4. After 72 h, cells were harvested, and protein levels were analyzed using western blotting. 18E6, E6AP, and FBXO4 were probed using their respective antibodies, and α-tubulin was used as a control. (**E**) 293E6AP K/O cells stably expressing either GFP alone or GFP-tagged 18E6 were transfected with siLuci (control) and siFBXO4. After 72 h, cells were harvested, and protein levels were analyzed using western blotting for each protein, as indicated. α-Tubulin was used as a loading control. (G and I) 293E6AP K/O cells were transfected with HA-tagged 18E6 or HA-tagged 11E6, together with myc-E6AP and Flag–FBXO4. After 24 h, cells were harvested and proteins levels analyzed using immunoblotting. Antibodies including anti-HA, anti-myc, and anti-Flag were used to detect 18E6 and 11E6, E6AP, and FBXO4, respectively. β-gal was used as loading control. (B, D, and F) Represent the statistical analysis of E6 protein levels normalized with control Luci from at least three independent experiments. *P value < 0.05 in HeLa, C41, and HEK293 E6AP K/O cells stably expressing GFP alone (control) and GFP 18E6, respectively. (H and J) Statistical analysis of the 18E6 and 11E6 in the presence of FBXO4 and absence of E6AP; data from at least three independent experiments. **P-value < 0.05, statistically quantified using Student’s *t*-test; error bars indicate the standard deviation of the mean.

Based on these results, we decided to focus on FBXO4, which is a substrate recognition component of SCF (SCF–Cul1–Fbox complex), an E3 ubiquitin ligase complex. This complex recognizes and mediates the ubiquitination and subsequent degradation of target proteins in a proteasome-dependent manner (18). First, we repeated the knockdown experiment in HPV-18-positive cell lines, HeLa and C41, to determine whether the knockdown of FBXO4 could rescue the levels of HPV-18E6 protein in the absence of E6AP. We reverse-transfected HeLa and C41 cells with the siRNAs against E6AP, FBXO4, and Luci as control. After 72 h, cells were harvested, and protein levels were analyzed using immunoblotting. As shown in Fig. 3A and C, siE6AP reduced the levels of E6 oncoprotein in both HeLa and C41 cell lines, as expected and in agreement with previous studies (15), whereas siFBXO4 had no apparent effect. However, silencing both FBXO4 and E6AP significantly increased the levels of E6, when compared with siE6AP, in both cell lines. We also repeated the knockdown experiment in cells stably expressing the GFP-tagged E6 in E6AP K/O cells. As shown in Fig. 3E, siFBXO4 increased the levels of GFP–18E6, compared with siLuci control, whereas no changes in the GFP protein levels were observed. These results suggest that FBXO4 targets E6 for degradation in the absence of E6AP, as the double knockdown of FBXO4 and E6AP leads to the stabilization of E6 protein levels.

Next, we validated these results on E6 degradation in the presence of FBXO4 in HEK293 E6AP K/O cells. We co-transfected these cells with plasmids expressing Flag-tagged FBXO4, HA-tagged 18E6, and HA-tagged E6AP. After 48 h, cells were harvested, and protein levels were analyzed using immunoblotting. As shown in Fig. 3G, 18E6 protein levels were greatly reduced in the presence of FBXO4 and were stabilized in the presence of E6AP, whether or not FBXO4 was expressed. Taken together, these results suggest that FBXO4 targets the degradation of E6 in the absence of E6AP, as transfecting E6AP back into the cells rescued the levels of E6 oncoprotein.

Since E6AP is also known to stabilize low-risk HPV E6 oncoprotein (19), we wanted to ascertain whether FBXO4 could induce the degradation of low-risk HPV E6 in the absence of E6AP. E6AP K/O cells were transfected with plasmids expressing 11E6, Flag-tagged FBXO4, and HA-tagged E6AP. After 48 h, immunoblot analysis showed that the protein levels of 11E6 decreased dramatically when expressed with FBXO4 alone, but were stabilized in the presence of E6AP, as shown in Fig. 3I, similar to what we observed in the case of HPV-18E6. Taken together, these results suggest that FBXO4 targets low- as well as high-risk HPV-E6 oncoproteins for degradation when E6AP is absent.

### FBXO4 interacts with HPV E6 oncoprotein

**Fig 4:**
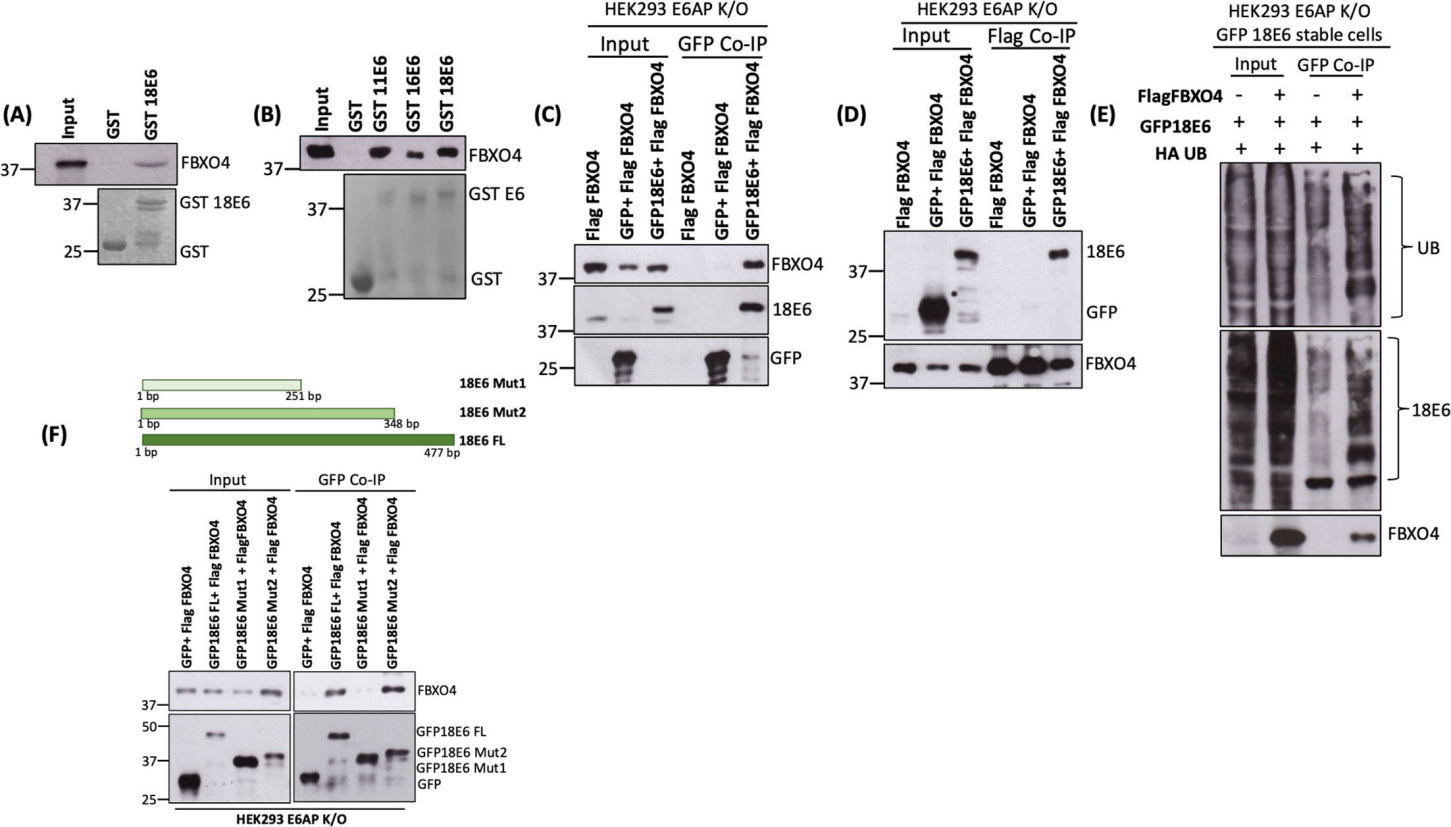
FBXO4 interacts with E6 oncoprotein specifically in the region from 251 to 348 bp: GST pull-down assays were performed using *in vitro* translated FBXO4 (**A**) and lysates of HEK293 E6AP K/O cells ectopically expressing Flag-tagged FBXO4 (5 µg), together with purified GST-tagged HPV-11E6, GST-tagged HPV-16E6, GST-tagged HPV-18E6, and GST alone as control. The top panels show the immunoblot analysis for FBXO4 protein probed using anti-FBXO4 antibody, and the lower panels show the Ponceau stain for different GST fusion proteins. (A and B) HEK293 E6AP K/O cells were transfected with plasmids expressing GFP alone or GFP-18E6, with or without the plasmid expressing Flag-tagged FBXO4 (5 µg). After 48 h, cells were treated with CBZ (20 nM) proteasome inhibitor, and then, after a further 5 h, cell extracts were analyzed by co-immunoprecipitation, using anti-GFP antibody (**C**) immobilized on agarose beads or anti-Flag immobilized on agarose beads (**D**). Proteins were analyzed using immunoblotting. FBXO4 was detected using anti-FBXO4 antibody; GFP-tagged 18E6 and GFP were detected using anti-GFP antibody. (**E**) 293 E6AP K/O cells stably expressing GFP-18E6 were transfected with HA-UB (2 µg), either with or without Flag-FBXO4 (5 µg). After 48 h, cells were harvested, and co-immunoprecipitation was performed using anti-GFP antibody followed by immobilization on agarose beads. Polyubiquitination patterns were detected, first using anti-HA and then anti-18E6 antibodies. Interaction of 18E6 and FBXO4 was confirmed using anti-FBXO4 antibody. (**F**) Mutational co-immunoprecipitation assay to map the region of E6 that interacts with FBXO4. HEK293 E6APK/O cells were transfected with plasmids expressing GFP-18E6 Mut1, GFP-18E6 Mut2, full-length GFP-18E6, and GFP alone (as control) together with Flag–FBXO4. Cell extracts were isolated after 48 h and co-immunoprecipitation performed using anti-GFP antibody immobilized on agarose beads. Interaction profiles for FBXO4 with different mutants of 18E6 were determined using immunoblot. Anti-GFP antibody was used to detect GFP-tagged proteins, and anti-FBXO4 was used to detect FBXO4.

To assess whether FBXO4 can interact with HPV E6, we first performed an *in vitro* GST pull-down assay, using a purified, bacterially expressed GST-18E6 fusion protein, together with *in vitro* translated FBXO4 protein. As shown in Fig. 4A, we found that FBXO4 bound GST-tagged 18E6, but not GST control. We next repeated the assay using GST-tagged HPV-11, -16, and -18 E6 proteins together with FBXO4 exogenously expressed in HEK293 E6AP K/O cells. FBXO4-expressing plasmid was transfected into the cells, and after 48 h, cells were lysed, and the cellular extract was incubated overnight at 4°C with GST-tagged HPV-11, -16, and -18 E6, and empty GST as a negative control, followed by immunoblot analysis. As shown in Fig. 4B, HPV-11E6 interacted most strongly with FBXO4, followed by HPV-18E6 and then HPV-16E6. We further validated this finding by performing a co-immunoprecipitation assay. We transfected HEK293 E6AP K/O cells with plasmids ectopically expressing Flag-tagged FBXO4, GFP-tagged 18E6, and empty GFP (as control). After 48 h, cells were treated with CBZ (20 nM) for 5 h. Cell extracts were isolated and incubated with anti-GFP antibody followed by co-immunoprecipitation on protein-G-conjugated agarose beads. The results, which are shown in Fig. 4C, show that GFP-tagged 18E6 interacts with FBXO4, while control GFP does not. We further verified this interaction in reverse, by performing the co-immunoprecipitation assay using Flag-tagged FBXO4 and probing the immunoblot for E6. As shown in Fig. 4D, GFP-18E6 was pulled down by FBXO4, while the control GFP was not. Taken together, these results indicate that 18E6 and FBXO4 interact with each other.

Next, we wanted to determine whether the presence of FBXO4 increased the ubiquitination of HPV-18E6. We transfected the GFP-tagged-18E6 E6AP K/O stable cell line with plasmids expressing HA-tagged ubiquitin and Flag-tagged FBXO4. After 48 h, cell extracts were harvested and incubated with anti-GFP overnight at 4°C followed by co-immunoprecipitation on protein-G-conjugated agarose beads. We observed increased ubiquitination of E6 in the presence of FBXO4 (Fig. 4E). This further suggests that FBXO4 binds HPV-18E6 and targets it for ubiquitination.

To investigate which region of 18E6 interacts with FBXO4, we first generated two different mutants of GFP-tagged 18E6, named GFP-18E6 Mut1 and GFP-18E6 Mut2 (shown in Fig. 4F). These mutants then were co-transfected into HEK293E6AP K/O cells, together with the Flag-tagged FBXO4-expressing plasmid. Full-length GFP-18E6 was used as a positive control and GFP alone as a negative control. After 48 h, cell extracts were prepared, and a co-immunoprecipitation assay was performed using anti-GFP. We found that both full-length GFP-18E6 and GFP18E6 Mut2, but not GFP alone or GFP18E6 Mut1, interacted with FBXO4 (Fig. 4F). These data indicate that the HPV-18E6 region from 251 to 348 bp is involved in the interaction with FBXO4.

### E6AP reduces the interaction between E6 and FBXO4

**Fig 5:**
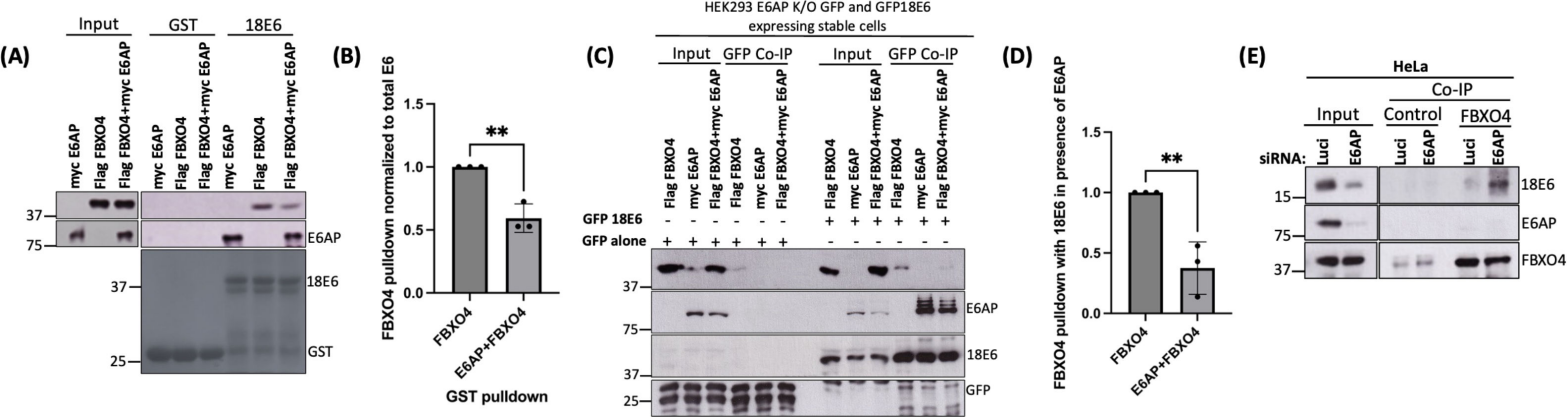
Interaction between FBXO4 and E6 is reduced in the presence of E6AP. (**A**) HEK 293 E6AP K/O cells were transfected with either HA-E6AP and Flag-FBXO4 or both. After 48 h, the cell extracts were isolated and incubated with GST alone (control) or GST-tagged 18E6. Binding was analyzed by western blotting. FBXO4 and E6AP were detected using anti-HA and anti-FBXO4 antibodies. Lower panel: the Ponceau stain for the GST and GST-tagged 18E6 proteins. (**C**) HEK 293 E6AP K/O cells stably expressing 18E6 GFP were transfected with plasmids expressing Flag-FBXO4 and HA-E6AP. After 48 h, cells were treated with CBZ (20 nM) for 5 h, cellular proteins were isolated and co-immunoprecipitated using anti-GFP antibody, followed by western blotting. FBXO4 and E6AP were probed using endogenous antibodies, respectively; GFP and 18E6 GFP were probed using an anti-GFP antibody. (**E**) HeLa cells were transfected with siRNA Luci (Control) and E6AP. After 72 h, cell lysates were co-immunoprecipitated using an anti-FBXO4 antibody and anti-GFP (as control) followed by immobilization on agarose beads. Analysis was performed using immunoblotting. 18E6, E6AP, and FBXO4 were detected using their respective endogenous antibodies. (B and D) Statistical analysis depicting the reduction in the interaction between FBXO4 and HPV-18E6 in the presence of E6AP from three independent experiments. The values for FBXO4 were normalized with the total E6 oncoprotein pulled down in each condition. **P-value < 0.05, statistically quantified using Student *t*-test; error bars indicate the standard deviation of the mean.

After confirming that 18E6 can bind FBXO4 in the absence of E6AP, we next were interested in ascertaining whether the presence of E6AP can perturb the interaction. We performed a series of binding assays between E6 and FBXO4 in the presence and absence of E6AP. We first performed a GST-pulldown assay as described above, using purified GST-tagged HPV-18E6 and GST alone (as control). As shown in Fig. 5A, when expressed individually with E6, both E6AP and FBXO4 can bind E6, but when all three proteins are co-expressed, the interaction between FBXO4 and E6 is markedly reduced. We also verified these results by repeating the co-immunoprecipitation assay by transiently expressing Flag-FBXO4 and HA-E6AP in HEK293 E6AP K/O cells stably expressing GFP- 18E6. Similar to the GST-pulldown findings, the presence of E6AP clearly reduced the interaction between E6 and FBXO4 (Fig. 5C).

We further validated these results in HeLa cells. We reverse transfected these cells with siE6AP or siLuci. After 72 h, cell extracts were isolated and incubated with anti-FBXO4 and anti-GFP antibodies overnight at 4°C, followed by immunoprecipitation. We observed a very weak interaction between E6 and FBXO4 in the presence of E6AP, which increased dramatically upon silencing E6AP (Fig. 5E), strongly suggesting that the presence of E6AP perturbs the interaction between E6 and FBXO4.

### Knockdown of FBXO4 and E6AP induces cell death in HPV-positive cervical cancer cells in a p53-dependent manner

**Fig 6:**
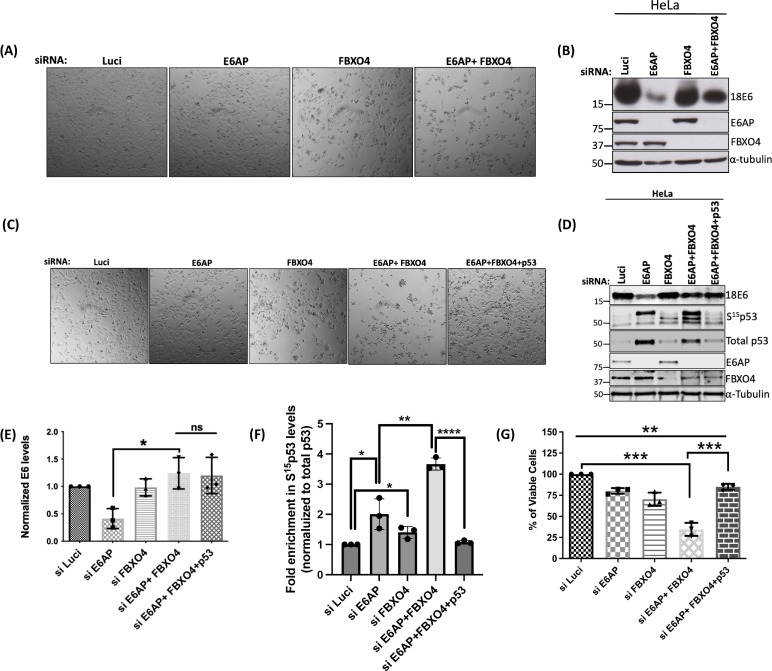
Ablation of E6AP + FBXO4 induces cell death in the cervical cancer-derived HeLa cell line in p53-dependent manner. HeLa cells were reverse transfected with siRNAs specific to E6AP, FBXO4, p53, and Luci (control) at a final concentration of 50 nM. After 72 h, live cell images were taken (A and C), and the cells were then harvested and protein levels analyzed using immunoblotting (B and D). 18E6, E6AP, S15 p53, p53, and FBXO4 were detected using the respective endogenous antibodies, and α-tubulin was used as loading control. (E and F) Statistical analysis depicting E6 and S15 phospho p53 levels from three independent experiments. The values for E6 were normalized with the loading control, and S15 p53 were normalized with total p53 levels.**, *P-value < 0.05; ns, non-significant, statistically quantified using Student *t*-test; error bars indicate the standard deviation of the mean. (**G**) Represents the XTT-based cell viability assay for HeLa cells. HeLa cells were reverse transfected with the siRNAs, as indicated, in a 96-well plate. After 72 h, cells were treated with XTT-labeling reagent followed by further incubation for 5 h. Cell viability was quantified using an ELISA plate reader at a wavelength of 490 nm. Statistical analysis was performed for each histogram using Student’s *t*-test. ***P-values < 0.001. Error bars represent the standard deviations from at least three independent experiments.

During our studies in HeLa cells, we observed that the silencing of E6AP together with FBXO4 not only increases the protein levels of HPV-18E6 (Fig. 6B) but also induces high levels of cell death, as shown in Fig. 6A. This was surprising, as we had expected that the rescue of E6 protein levels would lead to an increase in cell survival. To investigate a potential mechanism, with p53 being a strong candidate for inducing such apoptosis, we repeated the knockdown of E6AP + FBXO4 but also included transfection of an siRNA against p53. As shown in Fig. 6C, the downregulation of E6AP + FBXO4 induced a phenotype indicative of cell death (also shown in Fig. 6A), but surprisingly, this phenotype was reversed when we also silenced p53. However, the E6 protein levels remained unchanged in both conditions (Fig. 6D and E). It is noteworthy that the knockdown of both siE6AP and FBXO4 induces a greater degree of apoptosis compared to the knockdown of siE6AP alone, despite the lower overall levels of p53 observed in cells subjected to siE6AP + FBXO4 knockdown relative to cells with siE6AP knockdown alone. To elucidate the underlying mechanisms, we analyzed posttranslational modifications of p53 in the aforementioned samples. Intriguingly, we observed a substantial increase in the levels of phosphorylated p53 at serine 15 (S15 phospho p53), a marker indicative of p53-mediated apoptosis, in cells subjected to E6AP knockdown, with a modest elevation in cells subjected to FBXO4 knockdown. Moreover, the concurrent knockdown of both E6AP and FBXO4 resulted in over threefold increase in S15 phospho p53 levels, which decreases upon the knockdown of E6AP + FBXO4 + p53 (shown in Fig. 6D and F). Taken together, these results suggest that the knockdown of E6AP + FBXO4 increases the E6 protein levels and induces cell death in a p53-dependent manner. We wanted to validate these results with the XTT-based colorimetric assay for cell viability. The principle of this assay is based on the cleavage of the tetrazolium salt XTT in the presence of an electron-coupling reagent, producing a soluble colored formazan salt, associated with cellular metabolic activity (20). To perform this assay, we repeated the knockdown experiment as described earlier, but in a 96-well format. After 72 h, the analysis was performed. As shown in Fig. 6G, silencing of E6AP and FBXO4 individually slightly decreased the number of viable cells, when compared with the Luci control. However, when both E6AP and FBXO4 were knocked down, this effect was more pronounced, with the cell viability being reduced to about 25%. However, silencing p53 together with E6AP + FBXO4 reversed this effect, increasing the cell viability to above 75%. These results demonstrate that the reduction in cell viability upon silencing E6AP + FBXO4 is dependent on the presence of p53 in HeLa cells.

**Fig 7:**
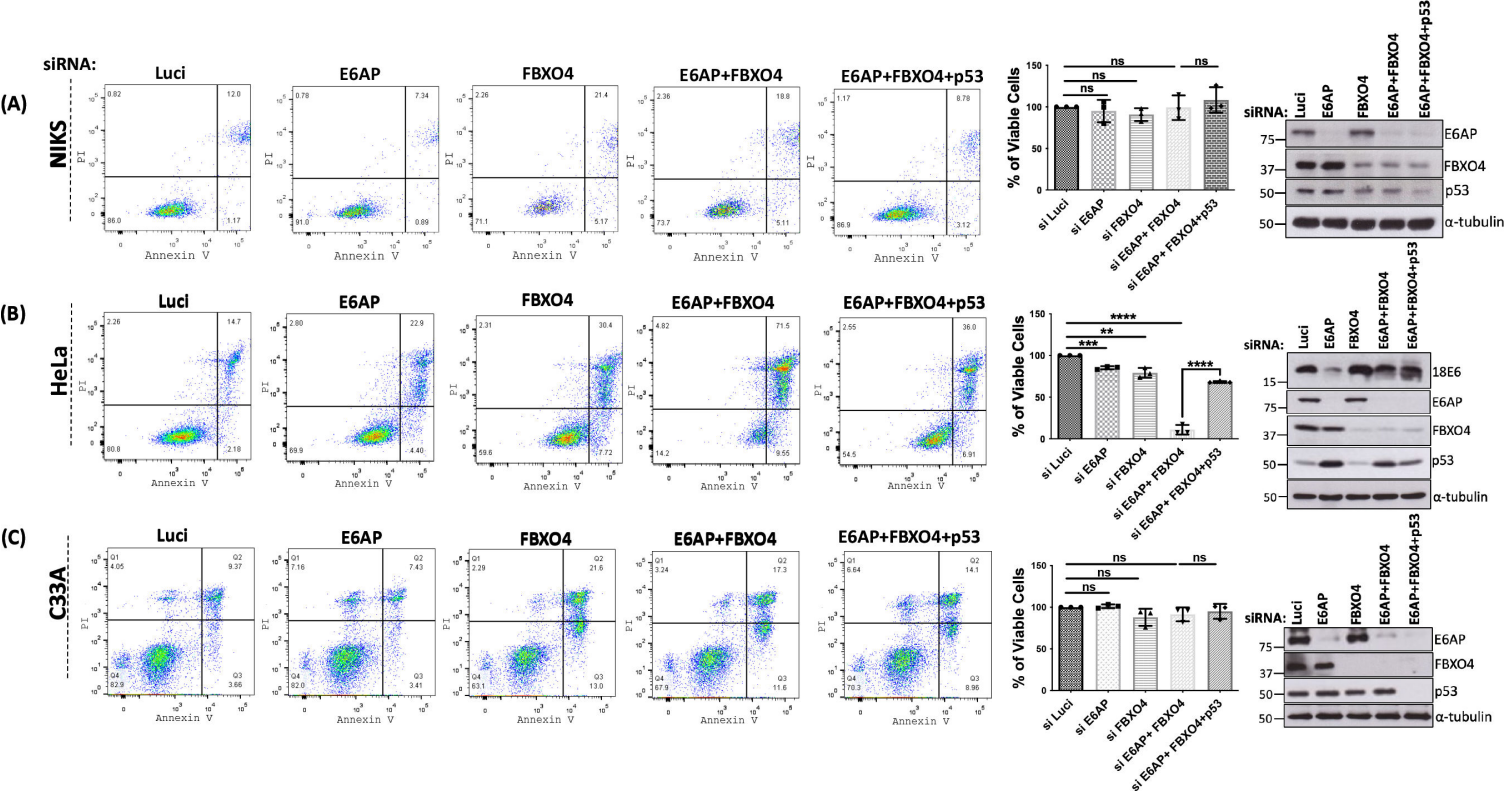
Depletion of E6AP and FBXO4 decreases cell viability in HeLa cells in a p53-dependent manner. NIKS (**A**), HeLa (**B**), and C33A (**C**) cells were reverse transfected with siRNAs against Luci (control), E6AP, FBXO4, and p53 at a concentration of 50 nM. After 72 h, cells were collected and evaluated for apoptosis using fluorescence-activated cell sorting (FACS) analysis (**A**). In the different quadrants, the percentage of cells were reported: viable cells, lower left quadrant (Q4); early apoptotic cells, bottom right quadrant (Q3); late apoptotic cells, top right quadrant (Q2); non-viable necrotic cells, upper left quadrant (A1). Scatter dot blots were plotted using FlowJo software. The middle lane represents the histogram showing the statistical analysis of the percentage of viable cells for NIKS, HeLa, and C33A. The last lane represents the western blot analysis showing the knockdown of all the proteins; E6AP, FBXO4, and p53; E6 protein levels were also checked in HeLa cells. α-Tubulin was used as a loading control.

**Fig 8:**
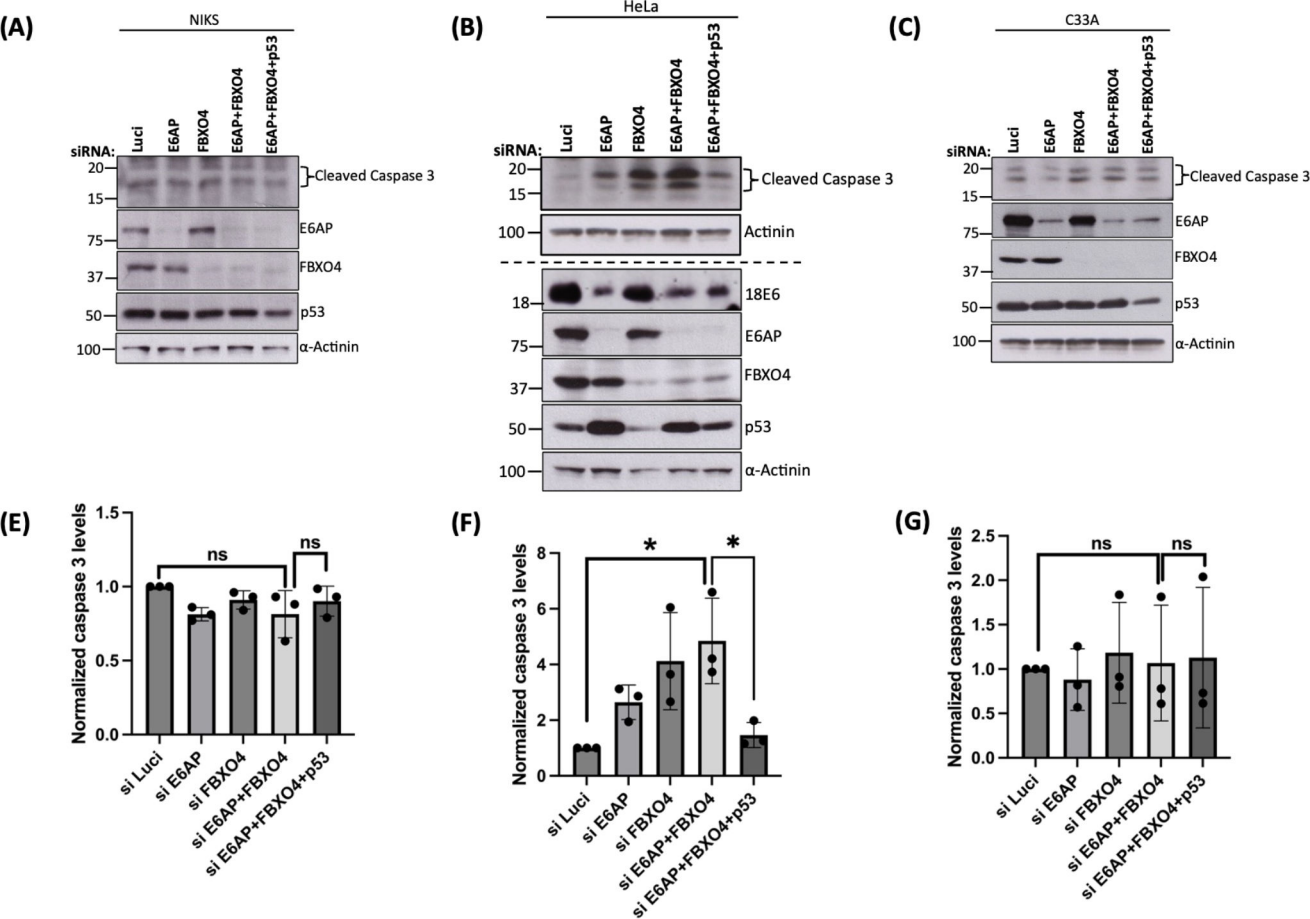
Knockdown of FBXO4 +E6 AP induces p53-dependent activation of caspase 3 in HPV-positive cancer cells. NIKS (**A**), HeLa (**B**), and C33A (**C**) cells were transfected with siLuci (control), siE6AP, siFBXO4, and sip53. After 72 h, cells were harvested, and immunoblot analysis was performed for all proteins as indicated in the figure using the respective antibodies, and α-actinin was used as loading control. The top blot in panel B represents the blot for the cleaved caspase 3 for all the samples, whereas the bottom blot shows the knockdown of all the proteins from the same samples run on different gels. (E, F, and G). Statistical analysis of cleaved caspase 3 in NIKS (**E**), HeLa (**F**), and C33A (**G**) with values normalized to loading control α-actinin from three independent experiments. *P-value < 0.05; ns, non-significant; statistically quantified using Student *t*-test; error bars indicate the standard deviation of the mean.

Having shown that the combined knockdown of E6AP and FBXO4 leads to dramatic cell death in a p53-dependent manner, we wanted to determine whether this effect is specific to HPV-positive cervical cancer cells. We performed apoptosis-based assays using the cell death markers Annexin-V and propidium iodide (PI) in three different cell lines, including NIKS (normal immortalized keratinocytes), HeLa (HPV-18 positive cervical cancer cells) and C33A (HPV-negative cervical cancer cells). In these cell lines, we repeated the knockdown experiment described in previous experiments. After 72 h, the cells were collected and labeled with PI and Annexin-V FITC, followed by flow cytometry. The results, which are shown in Fig. 7, indicated that the knockdown of E6AP in NIKS (Fig. 7A) and C33A (Fig. 7C) cells resulted in no significant differences in the percentage of viable cells, when compared to siLuci control, whereas in HeLa cells, silencing of E6AP significantly decreased cell viability (Fig. 7B), in agreement with the previous studies (21). We also found that the downregulation of FBXO4 from all the three cell lines decreased the percentage of viable cells, which is not unexpected as FBXO4 has been shown to be involved in the regulation of cancer cell growth (22). Intriguingly, when both E6AP and FBXO4 were silenced together in HeLa cells, the percentage of viable cells decreased dramatically compared with the Luci control, whereas there was no effect on the viability of NIKS and C33A under these conditions. In addition, the triple knockdown of E6AP + FBXO4 + p53 in HeLa cells significantly rescues the number of viable cells, when compared with siE6AP + FBXO4. Again, we observed no significant changes in the cell viability for the triple knockdown in NIKS or C33A cells. We validated the knockdown of all the proteins using immunoblotting, as shown in Fig. 7. In addition, we observed a marked increase in the levels of cleaved caspase 3 protein upon knockdown of E6AP + FBXO4 in HeLa cells, which was rescued upon the further knockdown of p53, while no such differences in the expression patterns of cleaved caspase 3 were seen in case of C33A and NIKS cell lines (Fig. 8). Taken together with the previous observations, this result indicates that the decrease in cell viability and increase in apoptosis upon silencing E6AP + FBXO4 is mediated by p53 and is specific to HPV-positive cancer cells.

## DISCUSSION

Human papillomaviruses (HPVs) have evolved several strategies to utilize the ubiquitin–proteasome system, via the E6 and E7 oncoproteins, to benefit the virus life cycle; this can, on rare occasions, also induce carcinogenesis (23–25). Both HPV E6 and E7 oncoproteins are unstable and have been reported to be degraded by the ubiquitin–proteasome system (24). Intriguingly, HPV E6 is stabilized by its interaction with the E6AP ubiquitin ligase. However, there is no information available about the ubiquitin ligases that regulate the stability and activity of HPV E6 in the absence of E6AP (15). We, therefore, became interested in identifying the ubiquitin ligase(s) involved in E6 degradation when E6AP is absent. To identify these, we performed a high-throughput siRNA library screen of approximately 534 human ubiquitin ligases in HEK293 E6AP K/O cells stably expressing GFP-tagged 18E6. We found a total of 11 ubiquitin ligases, the knockdown of which increased the GFP-18E6 fluorescence, and four ubiquitin ligases the knockdown of which was instead inhibitory.

To validate these findings, we examined the effects of the 11 siRNAs that increased GFP-E6 levels. Interestingly, in HeLa cells, of the 11 candidates, silencing of E6AP + FBXO4 gave the maximum increase in the 18E6 protein levels, followed by ablation of E6AP + FBXL19 and E6AP + TRIM69, and then E6AP + BAZ2B, compared with siE6AP alone. FBXO4 and FBXL19 are the F-box proteins that form functional complexes with Skp1 and CUL to generate the E3 SCF multi-subunit complex (26). TRIM69, a member of the tripartite motif (TRIM) family of proteins, is a RING-type E3 ubiquitin ligase, functionally known for its anti-viral defense against various viruses (27). BAZ2B belongs to the bromodomain gene family that encodes a protein involved in chromatin remodeling (28). Based on validation, we obtained in HeLa cells, and after finding a marked increase in 18E6 protein levels upon the knockdown of FBXO4 together with E6AP, we decided to concentrate our attention on this ubiquitin ligase for further analysis.

We first showed in degradation assay, performed in HEK293 E6AP K/O cells, that the ectopic expression of FBXO4 with GFP-18E6 decreased the levels of 18E6, suggesting that the presence of FBXO4 increases the degradation of 18E6 in the absence of E6AP. We also validated these results in cervical cancer-derived cell lines, HeLa and C4-1, and observed similar results. Our *in vitro* studies revealed that FBXO4 does not significantly alter HPV-16E6 protein levels (data not shown), unlike its effect on HPV-18E6 and -11E6. The reduced FBXO4-HPV-16E6 interaction in E6AP’s absence suggests alternative ubiquitin ligases may regulate HPV-16E6 degradation. However, the lack of a specific HPV-16E6 antibody limited our ability to validate these findings in endogenous systems. These results highlight the potential for type-specific regulation of HPV E6 proteins and underscore the need for further investigation. Our GST-pulldown and co-immunoprecipitation assays revealed a direct interaction between E6 and FBXO4, which is disrupted by E6AP. This suggests a complex interplay among these proteins, where E6AP competes with FBXO4 for E6 binding. We propose that E6’s higher affinity for E6AP results in steric hindrance, preventing efficient FBXO4–E6 interaction. This model explains our observation that FBXO4 targets E6 for degradation only in E6AP’s absence, thereby elucidating a novel mechanism of E6 regulation. Further structural and biochemical studies are required to fully characterize these interactions and their implications in HPV biology.

We also demonstrated that the combined knockdown of E6AP and FBXO4 not only rescued the levels of 18E6 but also significantly reduced cell viability in a p53-dependent manner. Further investigation into the posttranslational modifications of p53 revealed that E6AP knockdown resulted in increased levels of S15 phospho p53, while FBXO4 knockdown led to a modest increase. Remarkably, the concurrent knockdown of both proteins enhanced S15 phospho p53 levels by more than threefold. We found that the knockdown of p53, together with that of E6AP and FBXO4, reduced the S15 phospho p53 levels, rescued cell viability, without changing the levels of E6. It could be possible that an increase in the HPV E6 levels upon knockdown of E6AP + FBXO4 disrupts the cellular homeostasis, which in turn hyperactivates the p53-mediated apoptotic pathway leading to increased cell death in HeLa cells. These findings suggest that E6AP and FBXO4 may regulate apoptotic pathways through distinct or complementary mechanisms, emphasizing the crucial role of p53 activation in mediating cell death. It is noteworthy that FBXO4 has been shown to regulate several cellular substrates, including p53, that are involved in controlling cell cycle progression and DNA damage, cellular senescence, and tumor metabolism (29, 30). Additionally, Lee et al. showed that FBXO4 plays a crucial role in telomere maintenance, as its knockdown results in a reduction in telomere length and reduced cellular proliferation (22). HeLa cells have constitutively active telomerase to sustain their proliferation, which is consistent with the effect we observe. This regulatory role of FBXO4 might well explain the decrease in cell viability observed in the cells in which FBXO4 was knocked down.

We also found that silencing of E6AP + FBXO4 induces apoptosis by activating caspase 3 in HPV-positive HeLa cells in a p53-dependent manner but had no apparent effect in either NIKS or C33A cells, suggesting that this phenotype is specific to HPV-positive cells. In these experiments, we observed a decrease in the number of viable cells in HeLa cells, when FBXO4 expression was depleted.

In conclusion, our study identifies FBXO4 as a novel E3 ubiquitin ligase that targets HPV-18E6 for degradation in the absence of E6AP and highlights the complex interplay between E6AP and FBXO4 in modulating E6 levels, p53 activation, and cell viability. These findings provide new insights into the mechanisms by which HPV E6 exploits the cellular ubiquitin–proteasome system and open avenues for investigating other components that may target E6 for degradation or be utilized by E6 to target its substrates in the absence of E6AP. A recent study by Wang et al. (31) elucidates the structural basis of E6AP activation by HPV E6, revealing how E6 binding induces conformational changes in E6AP that promote dimerization and enhance substrate ubiquitination (31). Building on these findings, it could be possible that FBXO4 may serve as a secondary regulatory mechanism for E6 when not bound to E6AP, potentially targeting free E6 for degradation to maintain cellular homeostasis. This dual-control system could be critical for regulating E6 levels and activity, though the specific interplay between E6AP-mediated stabilization and FBXO4-directed degradation requires further investigation. Further research into these mechanisms may contribute to the development of novel therapeutic strategies targeting HPV-related malignancies.

## MATERIALS AND METHODS

### Cell culture

HeLa, C41, C33A, and HEK293 E6AP knockout cells were grown in Dulbecco’s modified Eagle’s medium (GIBCO, #31885-023) supplemented with 10% fetal bovine serum (GIBCO, #10270-106), penicillin–streptomycin (100 U mL^−1^), and glutamine (300 µg mL^−1^) (GIBCO, 10378-016) at 37°C in a humidified air incubator containing 10% CO_2_.

NIKS were grown in Ham’s nutrient mixture F-12 medium (GIBCO, #21765-029) supplemented with 5% fetal bovine serum (GIBCO, #10270-106), 0.4 µg/mL of hydrocortisone, 5 µg/mL of insulin, 10 ng/mL of EGF, 24 µg/mL of adenine and penicillin–streptomycin (100 U mL^−1^) at 37°C in a humidified air incubator containing 10% CO_2_

### Chemicals, inhibitors, and antibodies

The inhibitors and antibodies used for the experiments in this study were as follows: The proteasome inhibitor used was CBZ (MG132 Z-Leu-Leu-Leu-al, Sigma Aldrich # C2211).

Primary antibodies used were mouse monoclonal 18E6 (1:500, Santa Cruz, #sc365089) and mouse monoclonal p53 (1:2,000, Santa Cruz, #sc126), mouse monoclonal E6AP (1:500, BD Biosciences, #611416), mouse monoclonal α-tubulin (1:20,000, Cell Signaling Technology, #T5168), mouse monoclonal anti-FBXO4 (1:500, Santa Cruz, #sc376872), mouse monoclonal anti-Flag (1:1,000, Sigma Aldrich, #F3165), mouse monoclonal anti-myc antibody (1:1,000, Santa Cruz, #sc-40), rabbit polyclonal anti-GFP (1:2,000, Abcam, #ab6556), mouse monoclonal actinin (1:4,000, Santa Cruz, #sc17829), rabbit polyclonal anti-caspase 3 (1:500, CST, #9664S), mouse monoclonal β-Gal (1:4,000, Promega, #Z378B), rabbit polyclonal S15 p53 (1:500, Cell Signaling Technology, #9284), mouse monoclonal HA-tag-peroxidase (1:4,000, Sigma Aldrich, #H6533-1VL), followed by HRP-conjugated anti-rabbit (1:2,000, DAKO, #P0260) and anti-mouse secondary antibody (1:2,000, DAKO, #P0217).

### Plasmids and cloning

The plasmids used were as follows: pGWI myc-E6AP WT (15), and pCDNA His-LacZ (Invitrogen). pGWI-18 E6 and pcDNA 11E6 have been described previously (32). The GST, HPV-11E6 GST, HPV-16E6 GST, HPV-18E6 GST, fusion proteins have been described previously (14, 33–35). pcDNA3 Flag-tagged FBXO4 was a kind gift from Prof. J. Alan Diehl from the University of Pennsylvania, USA; HA-tagged UB has been described previously (36).

pHAHA-Empty GFP and pHAHA-GFP18E6 were made by sub-cloning GFP from peGFP C1 plasmid (Addgene, # #6084–1) using Nhe1 and BamH1 restriction sites into pHAHA empty vector (Addegene, #12617) (37). We then used pHAHA-Empty GFP to clone 18E6. To make pHAHA-GFP18E6, we first amplified 18E6 from pEGFP-18E6 plasmids, using EcoR1 and Sal1 restriction sites and then cloned it into the pHAHA-GFP vector. 18E6 GFP Mut1 mutant cloned into peGFP plasmid using restriction sites EcoR1 and Sal1 sites and 18E6 GFP Mut2 was generated using site-directed mutagenesis in peGFP18E6 plasmid.

The primer sequences used are as follows: GFP18E6_F, ATA GAA TTC ATG GCG CGC TTT GAG GAT C, and GFP18E6_R, CCT GTC GAC TTA TAC TTG TGT TTC TCT GC; GFP18E6 Mut1_F, CGCGAATTCATGGCGCGCTTTGAGGATCC, and GFP-18E6 Mut1_R, CGCGTCGACTTAAGAGTCTGAATAATGTCTTAA; and GFP18E6 Mut2_F,CCGTTGAATCCAGCATAAAAACTTAGACACC, and GFP-18E6 Mut2_R, GGTGTCTAAGTTTTTATGCTGGATTCAACGG.

### GST-pulldown and immunoprecipitation assays

HEK293 E6AP knockout cells were transfected with the appropriate plasmids and then harvested after 48 h in lysis buffer (50 mM HEPES [pH 7.4], 150 mM NaCl, 1 mM MgCl_2_, 1% Triton X-100), supplemented with protease inhibitor cocktail I (Millipore, #539131). Cellular extracts were incubated with GST fusion proteins immobilized on glutathione agarose for 2 h at room temperature. After exhaustive washes, bound proteins were detected by western blotting.

To perform the co-immunoprecipitation assay for FBXO4 and E6 interaction, HEK293 E6AP K/O cells stably expressing GFP and GFP-18E6 were transfected with Flag-tagged FBXO4 plasmid. After 48 h, proteasome inhibitor CBZ (20 nM concentration) was added to each sample for 5 h. After this incubation period, cells were harvested in lysis buffer, as above. Cell lysates were first incubated with rabbit polyclonal anti-GFP antibody (1-µg concentration) overnight at 4°C followed by incubating the antibody–cell lysate mix with protein G (Amersham Biosciences, #17-0618-02) for 2 h at room temperature. Beads were extensively washed, and protein analysis was performed using western blotting.

### Generation of GFP-tagged 18E6 and empty-GFP stable cell lines in HEK293 E6AP K/O cells

HEK293 E6AP K/O cells were transfected with plasmids expressing pHAHA-GFP empty and pHAHA-GFP18E6 containing a hygromycin-resistance gene (Thermo Fischer # 10687010) using the CaCl_2_ transfection protocol. After 24 h, cells were selected using hygromycin at a concentration of 200 µg/mL for 2 weeks, changing the media containing hygromycin antibiotic every 2 days. After 2 weeks, 10 cells per 10-cm petri dish were seeded in multiple plates, which were then allowed to form colonies for the next 2 weeks. Around 20 colonies were picked for each pHAHA-Empty GFP and pHAHA-GFP18E6, followed by validation of the protein expression using western blotting.

### siRNA library screening

The human cellular ubiquitin ligase siRNA library was purchased from Dharmacon containing ~598 target genes (siGENOME SMARTpools of four siRNAs against each gene). To perform the screening, siRNAs were transferred robotically from stock library plates to poly-L lysine-coated 384-well plates (Perkin Elmer), leaving two columns empty for the addition of all relevant controls, which includes buffer, non-targeting siRNAs, siRNA against UBC gene, and proteasome inhibitor CBZ (added at 67 h after transfection for a total of 5 h). siRNAs were reverse transfected into HEK293 E6AP GFP18E6 stable cells at a final concentration of 50 nM. After 72 h, paraformaldehyde (PFA) was added to each well without removing the media from the wells from the stock of 16% to give a final concentration of 4% for 15 min at room temperature. Cells were then washed twice with phosphate-buffered saline (PBS) and permeabilized using 0.5% Triton-X in PBS for 20 min at room temperature. After this, cells were washed thrice with PBS, and nuclei were stained using Hoechst 33342 and HCS CellMask Deep Red staining (Thermo Fisher Scientific #H32721). Images were acquired using an Operetta HTS microscope (PerkinElmer) at a magnification of ×20; a total of 15 images were acquired for each well and replicate.

Image analysis was performed using Columbus image analysis software (PerkinElmer). In particular, nuclei were segmented based on Hoechst 33342 staining, and the cellular region was segmented based on HCS CellMask Deep Red staining. The mean intensity of the GFP18E6 signal in the cellular region was calculated. Two independent biological replicates were run, and *Z*-score was calculated for both total nuclei number (cell viability) and GFP mean cellular intensity. Compounds reducing total cell number (*Z*-score ≤1.96; *P* ≤ 0.05) were excluded from further analysis. Compounds significantly increasing GFP mean cellular intensity (*Z*-score ≥1.96; *P* ≤ 0.05) in both replicates were followed up. The screening was performed at the ICGEB High-Throughput Screening Facility (https://www.icgeb.org/high-throughput-screening-equipment/).

### XTT-cell viability assay

Cells were reverse transfected with the relevant siRNAs in a 96-well plate in a final volume of 100-µL culture medium per well. After 72 h of incubation, 50 µL of the XTT labeling mixture (prepared by mixing 5 mL of XTT labeling reagent with 0.1 mL of electron coupling reagent) (Roche, #11465015001) was added to each well, followed by incubation for 5 h in a humidified chamber with 10% CO_2_ maintained at 37°C. After this incubation period, the formazan dye formed was quantitated using ELISA reader at a wavelength of 490. The measured absorbance directly correlates to the number of viable cells.

### Fluorescence-activated cell sorting (FACS)

First, cells were reverse transfected with different siRNAs in a six-well plate. After 72 h of incubation, cells were washed with PBS, trypsinized, and centrifuged at 1,500 rpm for 5 min. After this, cells were re-suspended in 1 mL of PBS, out of which 200 µL was taken to perform FACS analysis, and the rest was used to perform western blotting. The 200 µL cell suspension in PBS was centrifuged at 1,500 rpm for 5 min, and the pelleted cells were re-suspended in 500 µL of incubation buffer (composition: 10 mM HEPES, pH 7.4, 140 mM NaCl, 5 mM CaCl_2_) containing Annexin-V Fluos (10 µL per 500 µL of incubation buffer) (Roche, # 11828681001) and PI (10 µL from a stock of 50 µg/mL per 500 µL of incubation buffer) (Sigma Aldrich, #P4864) followed by incubation for 15 min at room temperature. The numbers of live and apoptotic cells under each condition were detected using FACS (BD FACS Celesta). Analysis of data was performed using FlowJo software.

### Ubiquitination assay

For ubiquitination assays, the relevant plasmids were transfected into cells, and after 48 h, cells were lysed in lysis buffer, as above, for 1 h on ice. The cell lysates were incubated with anti-GFP followed by conjugation on agarose beads (Sigma Aldrich, #F2426) overnight at 4°C, to pull down ubiquitin-conjugated protein. The beads were then washed thrice for 5 min with lysis buffer. The bound proteins were eluted in 2× sample buffer, and the polyubiquitinated E6 protein was detected using western blotting.

### siRNA transfection

Cells were seeded at a confluence of 30%–40% in a six-well plate, and after 24 h, they were transfected with siRNAs against E6AP (Dharmacon, SMARTPool #L-005137–00-0005), FBXO4 (Dharmacon, #J-012433-05-0005), Luciferase (Dharmacon, #D-002050-0-1-20), using Lipofectamine RNAMaxi (Life Sciences Technologies, #13778-150). After 72 h of incubation, cells were harvested and analyzed using western blotting.

## SUPPLEMENTAL MATERIAL

Table S1 mbio.02783-24-s0001.xlsx Log FC in GFP-tagged 18E6 intensity for all the ubiquitin ligases present in the library screening.

ASM does not own the copyrights to Supplemental Material that may be linked to, or accessed through, an article. The authors have granted ASM a non-exclusive, world-wide license to publish the Supplemental Material files. Please contact the corresponding author directly for reuse.
